# HIV Treatments Reduce Malaria Liver Stage Burden in a Non-Human Primate Model of Malaria Infection at Clinically Relevant Concentrations *In Vivo*

**DOI:** 10.1371/journal.pone.0100138

**Published:** 2014-07-02

**Authors:** Charlotte V. Hobbs, Jillian Neal, Solomon Conteh, Liam Donnelly, Jingyang Chen, Kennan Marsh, Lynn Lambert, Sachy Orr-Gonzalez, Jessica Hinderer, Sara Healy, William Borkowsky, Scott R. Penzak, Sumana Chakravarty, Stephen L. Hoffman, Patrick E. Duffy

**Affiliations:** 1 Laboratory of Malaria Immunology and Vaccinology, National Institutes of Health/National Institute of Allergy and Infectious Diseases, Rockville, Maryland, United States of America; 2 AbbVie Inc., North Chicago, Illinois, United States of America; 3 Division of Infectious Disease and Immunology, Department of Pediatrics, New York University School of Medicine, New York, New York, United States of America; 4 Clinical Center Pharmacy Department, Clinical Pharmacokinetics Research Laboratory, National Institutes of Health/National Institute of Allergy and Infectious Diseases, Bethesda, Maryland, United States of America; 5 Sanaria, Inc. Rockville, Maryland, United States of America; National University of Singapore, Singapore

## Abstract

We have previously shown that the HIV protease inhibitor lopinavir-ritonavir (LPV-RTV) and the antibiotic trimethoprim sulfamethoxazole (TMP-SMX) inhibit *Plasmodium* liver stages in rodent malarias and *in vitro* in *P. falciparum*. Since clinically relevant levels are better achieved in the non-human-primate model, and since *Plasmodium knowlesi* is an accepted animal model for the study of liver stages of malaria as a surrogate for *P. falciparum* infection, we investigated the antimalarial activity of these drugs on *Plasmodium knowlesi* liver stages in rhesus macaques. We demonstrate that TMP-SMX and TMP-SMX+LPV-RTV (in combination), but not LPV-RTV alone, inhibit liver stage parasite development. Because drugs that inhibit the clinically silent liver stages target parasites when they are present in lower numbers, these results may have implications for eradication efforts.

## Introduction

HIV and malaria geographically overlap. As more patients are managed for HIV exposure and infection, understanding HIV drug impact on malaria infection is important.

The World Health Organization (WHO) recommends HIV management with combination antiretroviral therapy (ARV), with first line therapy including a non-nucleoside reverse transcriptase inhibitor (NNRTI) and 2 nucleoside reverse transcriptase inhibitors (NRTIs) with few exceptions, and second line therapy including an HIV protease inhibitor (HIV PI) and 2 NRTIs [1]. We and others have previously shown that HIV PIs have a more potent effect against liver stage and asexual stage *Plasmodium* in rodent malaria models and in *P. falciparum in vitro* compared with NNRTIs [2]–[6]. Separately, many clinical studies have shown the antibiotic, trimethoprim-sulfamethoxazole (TMP-SMX), commonly used in HIV-exposed infants and HIV-infected patients in malaria endemic areas [1] can reduce clinical malaria burden [7], and we have previously shown that TMP-SMX blocks development of liver stage *Plasmodium* in rodent malaria models [4] and in *P. falciparum in vitro* [4]. However, whether LPV-RTV or TMP-SMX can reduce malaria burden through anti-liver stage effect in vivo in a model which better approximates human malaria infection, and drug treatment levels achieved in clinical practice, remains unknown. Moreover, clinical studies have not investigated the impact of HIV treatments on malaria liver stages. Drug effect on the liver stage is important to understand as during this stage there are no clinical symptoms, the number of infected human cells is low, and liver-stage killing drug effects may impact the acquisition of protective anti-malarial immunity. If medications used in HIV management can have a beneficial effect in reducing malaria burden through killing of liver stage parasites, these treatments may be tailored or timed to maximize those benefits.

The rhesus macaque-*P. knowlesi* infection model remains a preferred model used to study *Plasmodium* liver stages since it approximates the *P. falciparum* course of infection in young children and adults [8] and parallels *P. falciparum* in having a longer liver stage duration of parasite development, in contrast to the rodent malarias [9], [10]. The animal model for *P. falciparum* infection in the *Aotus spp.* monkey host is suboptimal because cycles of transmission through sporozoites are unreliably maintained [9] and the availability of *Aotus spp.* monkeys is also low [11]. Lastly and importantly, pharmacokinetics of the drugs tested *in vivo* in the rhesus macaque model very closely reproduces what is observed in humans on standard HIV treatments.

Here, we describe our investigations of the most potent antimalarial HIV PI, lopinavir-ritonavir (LPV-RTV), and the antimicrobial, TMP-SMX, each alone and in combination, on *P. knowlesi* liver stages. This model offers, for the first time, examination of these drug effects *in vivo* at clinically relevant concentrations, or concentrations that better approximate what is achieved in humans on standard dosing regimens. This paper also describes for the first time the use of cryopreserved *P. knowlesi* sporozoites, used as a surrogate for *P. falciparum in vivo* since liver stage duration is comparable in time [9], and which allows for reproducible and controlled sporozoites inoculum in this host.

## Methods

### Parasites and mosquitoes

To make cryopreserved sporozoites, *Plasmodium knowlesi* H strain parasitized red blood cells (NIH stock) were used to infect splenectomized, anesthetized monkeys (0.1 cc/kg 10% Ketamine IM). After infection, 30–160 *Anopheles dirus* crossed with *A. crascens* (Malaria Research and Reference Reagent Resource Center and Laboratory of Malaria Vector Research, NIAID) mosquitoes were fed on these monkeys. After feeding, mosquitoes not engorged with blood were removed. Purified *P. knowlesi* sporozoites were cryopreserved at Sanaria, Inc. using an unpublished method.

### Non-human Primates

The NIH facilities and laboratories where all of these studies were performed maintain a Public Health Service Assurance. The PHS policy mandates an Institutional Animal Care and Use Committee (IACUC) and the regulations describing its authority and responsibilities. Both the pharmacokinetic and drug testing studies were specifically approved by the National Institute of Allergy and Infectious Disease Intramural IACUC (NIAID IACUC, Permit “ASP LMIV 9E”), and all NIAID IACUC Guidelines were followed during both studies described. Furthermore, the NIH animal care and use program has opened itself to external review for independent accreditation by the Internationally recognized Association for Assessment and Accreditation of Laboratory Animal Care (AAALAC).

*Macaca mulatta* (rhesus macaque) monkeys of Indian origin and bred in the United States maintained in facilities accredited by the Association for Assessment and Accreditation of Laboratory Animal Care (AALAC) were used. All ancestors of these animals were imported with appropriate CITES permits. Animals are tested for *Mycobacterium tuberculosis* exposure quarterly and are Measles-immunized. For the pharmacokinetic study, all animals were female, were 5 years old, and ranged in weight from 3.12–3.62 kg (mean 3.49 kg) (Table 1). For the drug treatment study, 5 females and 3 males were included, age ranged from 5–7 years old (mean 5.625 years), weight ranged from 4.82–8.74 kg (mean 6.66 kg) (Table 2). Animals were observed at least twice per day with abnormalities reported to the American College of Laboratory Animal Medicine (ACLAM)-accredited staff veterinarian by the end of the observation period. NIAID has standard enrichment operating procedures which were followed for both of these studies, and details are as follows: animals were housed in cages with heights for the animals to stand erect comfortably with their feet on the floor. Diets included a high fiber monkey diet as well as a range of foods including fresh fruits, vegetables and nuts with water *ad libitum*. When there was room in the facility, animals were transferred to a play cage with swings at one-week intervals. Animals were provided with hanging and floor toys of differing shape, color, and texture, and rotated every 2 weeks, and checked for small pieces to prevent choking. Animals were pair housed to allow for social interaction, with perches in the cages allowing for exercise. The facility temperature range was 73°F–79°F, with humidity less than 70%, with regular light cycles maintained. In order to minimize the use of non-human primates, the study used animals who had experienced 1–8 prior *P. knowlesi* or *P. cynomolgi* infections previously (Table 2), the most recent of which preceded this study by 6 months. All malaria infections (for the prior and described study) were treated immediately upon smear detection. Animals were gavaged and infected under sedation with ketamine (0.1 cc/kg 10% Ketamine IM), with close monitoring for clinical and behavioral changes, and all efforts were made to minimize suffering. Our study endpoint was the detection of parasites on malaria smear at which time animals were immediately treated. No animals perished or were sacrificed during this study, and no adverse events were observed or reported.

**Table 1:** Animal Characteristics, Pharmacokinetic Study.^A^

Animal	Group/Treatment	Sex	Weight (kg)	Age (Years)
LX8A	LPV-RTV (sedated)	F	3.12	5
JMF	LPV-RTV+TMP-SMX (sedated)	F	3.6	5
JRH	LPV-RTV+TMP-SMX (awake)	F	3.62	5
JZC	TMP-SMX (sedated)	F	3.6	5

A Each animal had one prior infection, 2 years prior to the pharmacokinetic study, but animals represented in “Table 1” were only involved in the Pharmacokinetic Study, which did not involve infection.

**Table 2:** Animal Characteristics, Drug Treatment Study.^A^

Animal	Group/Treatment	Sex	Weight (kg)	Age (Years)	Number of Prior Infections
A8E008; EV3	Control	M; F	7.54;4.82	5;7	8; 2
AE032; GLM	LPV-RTV	F; F	5.94; 6.02	5; 6	8; 2
GLI; AE054	TMP-SMX	F; M	7.56; 8.74	6; 5	4; 8
G31^B^; A8E065	LPV-RTV+TMP-SMX	F; M	6.84; 5.8	6; 5	1; 8

A Sedated with 0.1 cc/kg 10% Ketamine IM.

B Prior infection with *P. cynomolgi*. All others were previously infected with *P. knowlesi*.

### Monitoring and treatment of malaria infections (Figure 1) for Drug Treatment Study

Giemsa stain thin smears and blood for PCR were collected every day from D5-D14, and then every other day until D28 (with infection on D0). Red cells (20×10^3^) per smear were examined before being declared negative. The first positive smear was detected on D10, which corresponded to a PCR value of 243 parasites/µl. Animals were treated when smear positive or by D28 with artesunate 8 mg/kg IV ×1, and chloroquine 20 mg/kg by gavage ×3 days [12].

### Drugs and Dosing (Figure 1)

Animals received 4 mg/kg of TMP + 20 mg/kg of SMX of commercially available suspension, and doses were derived from TMP-SMX prophylaxis for *Pneumocystis jirovecii*, although higher doses may be given [13]. Lopinavir-ritonavir (LPV-RTV) 50 mg/kg, based on the lopinavir component (provided by Abbvie, Inc.), was given. All drugs were administered by gavage. Two animals per group received TMP-SMX, LPV-RTV, or TMP-SMX and LPV-RTV together, or no drug (control). Drugs were administered in the morning in procedure rooms.

### Pharmacokinetic Study

**Figure 1:**
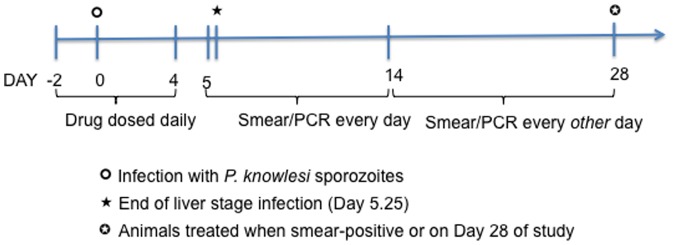
Schematic for Drug Treatment Study. Based on our available pharmacokinetic data and based on the assumption that 4–5 times half-life is required to reach steady state and then to eliminate drug in plasma [14], drugs levels were assumed to be at steady state (with dosing starting at D −2, and continued through D4) and mostly eliminated by the end of *P. knowlesi* liver stages (last dose given at 32 hr, and liver stages last about 5.25 days, [9]). On D −2, animals were infected IV with 2,500 cryopreserved *P. knowlesi* sporozoites. Smears and dried blood spots for PCR were collected every day from D5–14 and every other day from D14–D28. Animals were treated if parasites were detected on smear or on D28 (end of study) if smear negative throughout the study.

In a separate study performed to establish pharmacokinetic parameters prior to undertaking the Drug Treatment study (Figure 1), one monkey each was given a single dose of LPV-RTV, TMP-SMX, or both drugs (the combination shall be referred to as “LPV-RTV+TMP-SMX”), with and without anesthesia to determine whether anesthesia affected drug levels. Animals had complete metabolic profiles sampled prior to and at 24 hr post dose administration to ensure the absence of underlying illness (pre-study) or drug toxicity (post-study). Samples were collected at 0, 1, 2, 4, 6, 8, 12, and 24 hr post drug-adminstration.

TMP, SMX, LPV and RTV concentrations were simultaneously determined in monkey plasma samples by AbbVie Inc., using protein precipitation with acetonitrile to separate the components of interest, followed by HPLC-MS/MS quantitation. The concentration of each sample was calculated by least squares linear regression analysis of the peak area ratio of the spiked animal plasma standards versus concentration. Peak plasma concentrations (C_max_) and the time to peak plasma concentration (T_max_) for each compound were read directly from the plasma concentration data for each animal. The plasma concentration data were submitted to multi-exponential curve fitting using WinNonlin Software. The area under the plasma concentration-time curve from 0–24 hr after dosing (AUC_0–24_) was calculated using the linear trapezoidal rule for the plasma concentration-time profiles. (Tables 3 and 4)

**Table 3:** Pharmacokinetics of Sulfamethoxazole and Trimethoprim in Rhesus Monkeys.

		Sulfamethoxazole				Trimethoprim			
ID	Dosing Regimen	C_max_ (µg/mL)	T_max_ (hr)	T_1/2_ (hr)	AUC_0–24_ (µg*hr/mL)	C_max_ (µg/mL)	T_max_ (hr)	T_1/2_ (hr)	AUC_0–24_ (µg*hr/mL)
LK8A	LPV-RTV^A^								
JMF	LPV-RTV+TMP-SMX^A^	38.0	4.0	9.6	792	0.16	4.0	7.5	1.73
JRH	LPV-RTV+TMP-SMX^B^	57.7	8.0	7.1	950	0.13	4.0	8.1	2.23
JZC	TMP-SMX^A^	63.2	1.0	6.4	1013	0.30	1.0	2.6	1.46
N/A	TMP-SMX (human historical data)^1^^,^ 2	40–60^1^	1–4	6–12	1160^2^	1–2^1^	1–4	8–10	32.6^2^

A Sedated with 0.1 cc/kg 10% Ketamine IM.

B Awake.

1 Data obtained from a dose of 160 mg trimethoprim and 800 mg sulfamethoxazole (Patel et al. Clin Pharmacokinet 1980;5:405–423.

2 Data obtained from a dose of 5 mg/kg trimethoprim and 25 mg/kg of sulfamethoxazole iv (Dudley et a. Antimicrob Agents Chemother 1984;26:811–814).

**Table 4:** Pharmacokinetics of Lopinavir-ritonavir in Rhesus Monkeys.

		Lopinavir				Ritonavir			
ID	Dosing Regimen	C_max_ (µg/mL)	T_max_ (hr)	T_1/2_ (hr)	AUC_0–24_ (µg*hr/mL)	C_max_ (µg/mL)	T_max_ (hr)	T_1/2_ (hr)	AUC_0–24_ (µg*hr/mL)
LK8A	LPV-RTV^A^	10.3	12.0	nf	170.7	3.6	8.0	nf	37.8
JMF	LPV-RTV+TMP-SMX^A^	9.4	24.0	nf	105.8	0.8	24.0	nf	11.1
JRH	LPV-RTV+TMP-SMX^B^	10.9	6.0	nf	159.9	3.4	6.0	nf	23.2
JZC	TMP-SMX^A^								
N/A	LPV-RTV (human historical data)^1^	7.06	3–6	5.2	95	0.610	3–6	5.12	4.31

A Sedated with 0.1 cc/kg 10% Ketamine IM.

B Awake.

1 Ibarra M, Fagiolino P, Vázquez M, Ruiz S, Vega M, Bellocq B, Pérez M, González B, Goyret A. Impact of food administration on lopinavir-ritonavir bioequivalence studies. Eur J Pharm Sci. 2012 Aug 15;46(5):516–21.

### Assumptions for Drug Treatment Study (Figure 1)

Based on our available pharmacokinetic data and based on the assumption that 4–5 times half-life is required to reach steady state and then to eliminate drug in plasma [14], drugs levels were assumed to be at steady state (with dosing starting at D −2, or two days prior to infection, and continued through D4) and mostly eliminated by the end of *P. knowlesi* liver stages (last dose given at 32 hr prior the end of the liver stages, which last about 5.25 days, [9]). It is acknowledged that pharmacokinetic parameters from a limited number of animals may not be representative of the entire population of rhesus macaques. On D −2, animals were infected IV with 2,500 cryopreserved *P. knowlesi* sporozoites (Figure 1), determined to be the minimum number of cryopreserved sporozoites needed to reliably infect 100% of monkeys with a time to detection of parasites in blood of <10 days (Chakravarty and Hoffman, unpublished data).

### Real Time PCR

To identify *P. knowlesi* parasites blood samples, real time, quantitative PCR was used as previously described, with few modifications [15]. Briefly, Trizol (Invitrogen)-preserved RNA was isolated using a standard chloroform extraction protocol and an RNeasy Micro Kit (Qiagen) and then a QuantiTect Multiplex RT-PCR Kit (Qiagen) were used, with cycling conditions as follows: 50°C for 20 min, 95°C for 15 min, 50 cycles of 94°C for 45 sec, 60°C for 45 sec. Genus- and species-specific primers [15], [16] were used. An internal PCR control primer and probe sequences (IPC Primer sequences: Forward: 5′-GTT AAG GGA GTG AAG ACG ATC AGA-3′, Reverse: 5′-AAC CCA AAG ACT TTG ATT TCT CAT AA-3′ IPC Probe sequence: 5′-CTC TCC GGA GAT TAG AACTCT TAG ATT GCT-3′) were designed to amplify an internal control RNA standard. Calculated PCR sensitivity was 0.00019 parasites/µl. An increase in the time to detection of parasites in the blood by PCR was used to deduce reduced liver stage burden [17].

### Study Endpoints

If animals remained negative by smear, they were treated empirically on D28 (end of the study period). Statistics are descriptive [18].

## Results

TMP-SMX and LPV-RTV dosing as used are non-toxic and generate levels that are approximately what is achieved in patients on standard treatment and prophylaxis regimens (Tables 3 and 4).

**Figure 2:**
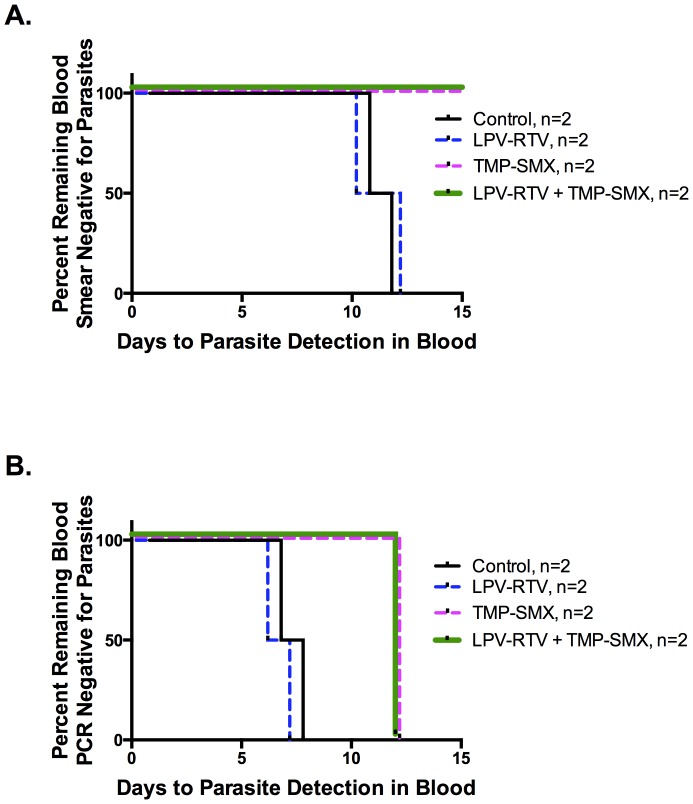
Trimethoprim-Sulfamethoxazole, and Lopinavir-ritonavir when used with Trimethoprim-Sulfamethoxazole, but not Lopinavir-ritonavir alone, at clinically relevant concentrations inhibits *P. knowlesi* liver stage parasites as reflected in absence of parasites in the blood by smear for 28 days (A) and prolonged time to PCR detection (B). To ensure drug steady state drug and 100% infection, rhesus (*Macaca mulatta*) monkeys were dosed starting 2 days prior to (D −2) through D4 post infection (with infection defined as D0) with 2,500 purified, cryopreserved *P. knowlesi* H strain sporozoites IV. Pharmacokinetic modeling used in this experiment predicted all drugs would be reduced to non-active levels prior to parasite emergence from the liver. Animals had Giemsa-stained smear and blood obtained from ear pricks until D28. Prolonged time to detection of parasites in blood was used to gauge liver stage parasite killing. (A) Control- and LPV-RTV-treated animals had positive smears on D10 and 11, and D10 and 12, respectively, but animals treated with TMP-SMX or LPV-RTV+TMP-SMX never became blood smear positive through D28, suggesting reduction of liver stage parasite burden. These findings were confirmed using (B) PCR for detection of parasites in blood (sensitivity, 0.00019 parasites/µl): parasites were detected earlier by PCR on D6 and 7 in controls and LPV-RTV (only)-treated animals, but on D12 in TMP-SMX only or in LPV-RTV+TMP-SMX combination-treated animals. Thus, LPV-RTV had no effect on the time to detection of parasites in blood, but TMP-SMX and LPV-RTV+TMP-SMX-treated animals had significant delays in detection of parasites in the blood, reflecting a reduced liver stage parasite burden.

By smear, Control- and LPV-RTV-treated animals were positive by D10 and 11, and D10 and 12, respectively, but animals treated with TMP-SMX or LPV-RTV+TMP-SMX remained negative through D28 (Figure 2A). Thus, blood stage parasitemia in these animals remained below the level of smear detection. Parasites were detected by PCR in both control and LPV-RPV-treated animals on D6 and 7. Thus, LPV-RTV alone had no effect on the time to detection of parasites in blood. In contrast, both TMP-SMX- and LPV-RTV+TMP-SMX-treated animals did not have detectable parasites by PCR until D12 (Figure 2B).

## Discussion

We have demonstrated that TMP-SMX and LPV-RTV+TMP-SMX-combination-treated animals had no detectable parasites by smear and prolonged time to PCR detection of parasites in blood, suggesting a reduction of liver stage parasite burden in the *P. knowlesi* rhesus macaque model. In contrast, LPV-RTV alone did not inhibit liver stages. This is the first evaluation of LPV-RTV and TMP-SMX *in vivo* in non-human primates against liver stage *P. knowlesi*, and all drug levels approximate clinically relevant concentrations.

We show TMP-SMX has a *P. knowlesi*-liver stage killing effect, as reflected in the increase in time to detection of parasites in blood. It is less likely that delayed parasite detection in TMP-SMX- (and LPV-RTV+TMP-SMX-treated animals) contributed to killing of blood stage parasites since the pharmacokinetic modeling dosing used in this experiment predicted drugs would be reduced to mostly non-active plasma levels prior to parasite emergence from the liver (see Tables 3 and 4, and [9], [14]). Of note, our pharmacokinetic study corroborates prior observations that TMP and SMX are each eliminated faster in rhesus monkeys compared with humans [19], which could account for why the AUC in our study, especially for TMP, is lower than what has been reported historically in humans (Table 3). Also, these data are consistent with our previous report that TMP-SMX delays liver stage parasite development in rodent malarias and in *P. falciparum in vitro* [2], [4], and other antifolates have such activity against *P. falciparum* in vivo [20], [21]. Delayed development of *Plasmodium* liver stages *in vitro* and *in vivo* has been observed with other antifolates, specifically, with *P. knowlesi* and *P.cynomolgi* [18], [22], [23], a *Plasmodium* which is studied experimentally in rhesus macaques also. It is likely that transmission intensity, as well as antifolate resistance, may contribute to the degree to which TMP-SMX reduces liver stage malaria burden in the field. Indeed, other studies have noted varying degrees of TMP-SMX efficacy in reducing clinical malaria, and these factors have been cited previously as being important in determining magnitude of effect [24].

We detected parasites by PCR and smear in controls and in LPV-RTV-treated groups but by PCR only for the TMP-SMX and the LPV-RTV+TMP-SMX groups likely because animals used in this study had had experienced *P. knowlesi* infection before. Blood stage immunity that controlled infection once and if parasites emerged from the liver may have developed in these animals from prior infections. However, our controls becoming positive earlier suggest that this immunity would not have affected liver stage parasite development.

As for LPV-RTV, our previous work has demonstrated that HIV PIs have anti-liver stage activity in *P. falciparum in vitro* [2]. However, *P. knowlesi* is a different parasite from *P. falciparum*, and this alone may explain the varied drug efficacy we have observed. Indeed, in another study, the less commonly used HIV protease inhibitor, indinavir, exhibited killing activity against *P. knowlesi* in rhesus macaques, although this effect was against asexual blood stage parasites [25]. The combination of LPV-RTV+TMP-SMX likely resulted in prolonged time to the detection of blood stage parasites due to TMP-SMX, since animals treated with LPV-RTV alone became positive on the same days as controls.

This study, importantly, also represents the first use of cryopreserved IV *P. knowlesi* sporozoites, which when injected at a 2,500 IV inoculum generates 100% patent blood stage infection. We believe that this is comparable in infectious inoculum to what would be observed with mosquito-host infection, and allows for controlled assessment of drug effects on liver stage parasites. Rodent malaria data shows that a few hundred sporozoites are injected by each mosquito in a time- and location-dependent manner (i.e., where on the body mosquitoes feed), with approximately half remaining in the skin, and the rest migrating to blood or lymphatic vessels [26]. Additionally, cryopreservation of sporozoites results in the loss of 85% of sporozoite inoculum, leaving approximately 375 viable sporozoites that are injected IV (Chakravarty and Hoffman, unpublished data). While the difference in species and route of administration may also affect the number of sporozoites that progress to the circulatory system and travel to the liver, we still are effectively administering only a few hundred infectious sporozoites per injection, comparable to what a mosquito would inject [26].

Field studies are required to validate whether PIs and TMP-SMX offer benefit in reducing clinical malaria through an anti-liver stage effect. If any such benefits are found, HIV treatments may be optimized for HIV-infected or exposed patients in malaria-endemic areas.
